# Video-assisted pulmonary metastectomy is equivalent to thoracotomy regarding resection status and survival

**DOI:** 10.1186/s13019-021-01460-8

**Published:** 2021-04-15

**Authors:** Till Markowiak, Beshir Dakkak, Elena Loch, Christian Großer, Monika Klinkhammer-Schalke, Hans-Stefan Hofmann, Michael Ried

**Affiliations:** 1 Department of Thoracic Surgery, University Medical Center Regensburg, Franz-Josef-Strauß-Allee 11, 93053 Regensburg, Germany; 2 Department of Thoracic Surgery, Hospital Barmherzige Brüder Regensburg, Regensburg, Germany; 3 Tumor Center, University Institute of Quality Assurance and Health Services Research, Regensburg, Germany

**Keywords:** Metastectomy, Video-assisted thoracoscopic surgery, Lung metastases, Pulmonary metastases, Prognosis

## Abstract

**Background:**

Surgical resection of pulmonary metastases leads to prolonged survival if strictly indicated. Usually, thoracotomy with manual palpation of the entire lung with lymph node dissection or sampling is performed. The aim of this study was to evaluate the role of video-assisted thoracoscopic surgery (VATS) in pulmonary metastectomy with curative intent.

**Methods:**

In this study, all patients with suspected pulmonary metastasis (*n* = 483) who visited the Center for Thoracic Surgery in Regensburg, between January 2009 and December 2017 were analysed retrospectively.

**Results:**

A total of 251 patients underwent metastectomy with curative intent. VATS was performed in 63 (25.1%) patients, 54 (85.7%) of whom had a solitary metastasis. Wedge resection was the most performed procedure in patients treated with VATS (82.5%, *n* = 52) and thoracotomy (72.3%, *n* = 136). Postoperative revisions were necessary in nine patients (4.8%), and one patient died of pulmonary embolism after thoracotomy (0.5%). Patients were discharged significantly faster after VATS than after thoracotomy (*p* < 0.001). Complete (R0) resection was achieved in 89% of patients. The median recurrence-free survival was 11 months (95% confidence interval 7.9–14.1). During follow-up, eight (12.7%) patients in the VATS group and 42 (22.3%) patients in the thoracotomy group experienced recurrence (*p* = 0.98). The median overall survival was 61 months (95% confidence interval 46.1–75.9), and there was no significant difference with regard to the surgical method used (*p* = 0.34).

**Conclusions:**

VATS metastasectomy can be considered in patients with a solitary lung metastasis. An open surgical approach with palpation of the lung showed no advantage in terms of surgical outcome or survival.

## Background

Pulmonary metastases often represent a manifestation of an advanced systemic disease. In addition to the induction of chemotherapy, surgical metastasectomy can also be a therapeutic option. In most cases, it is unlikely that metastectomy as a local measure will eradicate the cancer, and the lack of prospective studies has led to criticism of the procedure [1, 2]. However, resection of pulmonary metastases can be attempted in selected patients within a curative treatment concept [3]. In 1965, Thomford postulated principles for indications for pulmonary metastectomy that are essentially still valid today: technical resectability, a tolerable general and functional condition, a controlled primary tumour and the absence of extrathoracic lesions are preconditions to perform potentially curative resection [4].

To date, no prospective studies exist. The first prospective randomized trial on the impact of pulmonary metastectomy for colorectal cancer, the PulMiCC trial (Pulmonary Metastectomy versus Continued Active Monitoring in Colorectal Cancer), was stopped last year due to faltering recruitment [5]. However, good retrospective survival data have been reported after metastectomy for several tumour entities. For example, 5-year survival rates of 39–68% have been reported for colorectal cancer patients and 36–47% for renal cell carcinoma patients [6–10].

The completeness of resection has been identified as the most important factor concerning long-term survival, regardless of the primary tumour [11–13]. While at present, video-assisted thoracoscopic surgery (VATS) has replaced open thoracotomy in many indications, curative metastectomy is still commonly performed via an open procedure. In contrast to VATS, thoracotomy offers the advantage that the entire lung can be manually palpated intraoperatively, providing additional diagnostic opportunity with regard to possible occult metastases. Intraoperative palpation revealed malignant pulmonary nodules in 20% of patients whose nodules were not detected by preoperative computed tomography (CT) in a prospective randomized study in 2011 [14]. Ludwig et al. demonstrated that 6.9% of patients with a preoperative solitary metastasis had additional histologically confirmed metastases after thoracotomy and manual palpation. In patients with multiple preoperative metastases, the proportion was approximately one-quarter (27.4%) [15]. For this reason, thoracoscopic procedures are currently recommended only for diagnostic and palliative metastectomy [16].

Whether an additional manual examination justifies the considerably greater trauma caused by thoracotomy remains unclear, especially considering the increasing resolution of imaging and thus the better detection of small pulmonary nodules [17]. Therefore, the objective of this study was to evaluate the role of VATS in pulmonary metastectomy with curative intent.

## Methods

### Study population

**Fig. 1:**
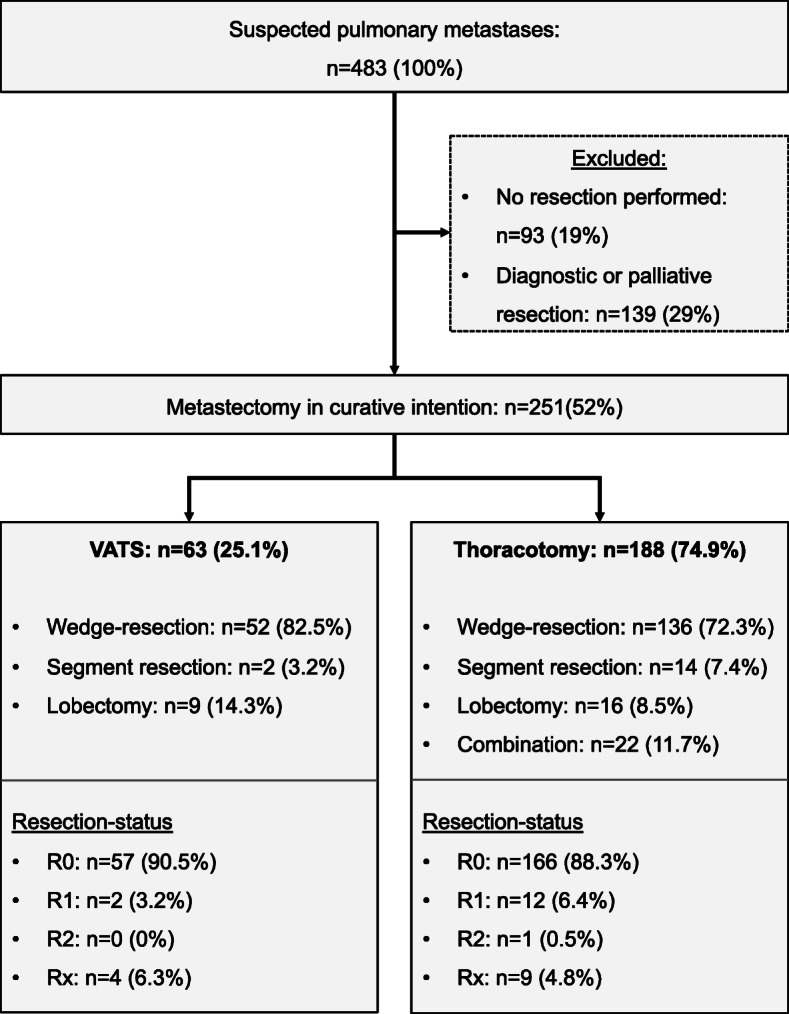
Flow-chart of the study population

In this study, all patients with suspected pulmonary metastases (*n* = 483) at the University Medical Center Regensburg and the Hospital Barmherzige Brüder Regensburg between January 2009 and December 2017 were analysed retrospectively. Before data collection, consent was obtained from the Ethics Committee of the University of Regensburg. Patients who received palliative, diagnostic or no resection were excluded from the study (*n* = 232) (Fig. 1).

In patients who were to undergo metastectomy with curative intent, either VATS or thoracotomy was performed. The surgical reports were analysed according to the resection procedure applied. The pathology reports were examined for histology of the tumour, the resection status (R0, R1, R2, and RX) and, in the case of VATS, the distance between the staple line and the tumour.

In January 2019, a follow-up examination was carried out at the regional Tumor Center, University Institute of Quality Assurance and Health Services Research, Regensburg. The resulting data were used to assess overall survival (OS), recurrence-free survival (RFS) and necessary re-operations due to pulmonary recurrence.

### Surgical approach

Patients in the thoracotomy group received standard anterolateral thoracotomy, usually in the 5th intercostal space. After insertion of a spreader and palpation of the nodule, a lung-sparing wedge or anatomical resection was performed. Lobectomy was necessary in patients with an unfavourable central location of the nodule after considering respiratory function. After resection, the entire remaining lung was palpated manually for coexistent, occult pulmonary nodules. Subsequently, systematic lymph node dissection or lymph node sampling was performed. In the case of suspected lymph node metastases (cN1/2), lymph node dissection was performed (the node level conformed to the primary lung cancer).

Access for VATS metastectomy was usually achieved via 2–3 ports. Depending on the location of the focus, wedge or anatomical resection was performed. Systematic manual palpation of the entire lung was not feasible in these cases.

### Study endpoints

The primary endpoints of this study were the rate of postoperative complications and in-hospital mortality. Secondary endpoints included the postoperative resection status, postoperative lymph node status and RFS as well as OS. Furthermore, we analysed the rate of re-operations due to pulmonary recurrence.

### Statistical analysis

The data were first collected in tabular form using Excel, version 15.0 (Microsoft Corporation, Redmond, WA, USA). For pseudoanonymization purposes, each patient was assigned a number. The statistical analysis was performed using IBM SPSS Statistics, version 24 (IBM Corporation, Armonk, NY, USA). Demographic data are summarized as numbers and percentages. The chi-squared test was used to analyse categorical variables between groups. If the minimum expected cell size assumption for application of the chi-squared test did not hold, Fisher’s exact test was used. The Kaplan–Meier method and the log-rank test were used for the survival analysis. A multivariate Cox regression model was generated to identify independent risk factors for mortality. A *p*-value < 0.05 was considered statistically significant.

## Results

### Patient characteristics

Between January 2009 and December 2017, 251 patients underwent pulmonary metastectomy with curative intent in the Center for Thoracic Surgery in Regensburg. A total of 232 patients were excluded from the study because surgery was not performed within the mentioned period, they received diagnostic/palliative metastectomy or histology did not confirm the suspected metastasis (Fig. 1).

**Fig. 2:**
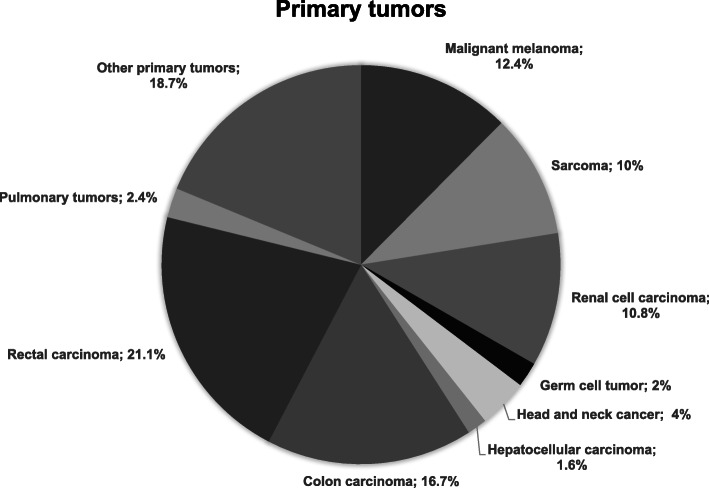
Primary tumor entities

The mean patient age was 61.5 ± 12.3 years, and most patients were male (*n* = 166; 66.1%). In 25.1% (*n* = 63) of patients, metastectomy was performed via VATS, while in 74.9% (*n* = 188) of patients, metastectomy was performed via thoracotomy. In both the VATS and thoracotomy groups, metastases were mostly solitary (85.7 and 55.3%). Colorectal cancer (rectal 21.1%, colon 16.7%) represented the majority of the primary tumour entity. At 12.4%, metastases of malignant melanoma were also frequent (Fig. 2). The primary tumour and metastases were diagnosed separately (metachronic) in more than 90% of cases, and in these patients, the disease-free interval was between 47 and 57 months. This interval was significantly longer in the VATS group (*p* = 0.022). Approximately two-thirds of the patients had pulmonary metastases only (Table 1).

**Table 1:** Patient characteristics, intra- and postoperative outcome

	VATS (***n*** = 63; 25.1%)	Thoracotomy (***n*** = 188; 74.9%)	***P***-value
Age (mean ± SD) [years]	64.9 ± 11.9	60.4 ± 12.2	0.93
Male gender; n (%)	42 (66.7%)	124 (66%)	0.92
Metastasis			
- Solitary; n (%)	54 (85.7)	104 (55.3)	< 0.001*
- Multiple; n (%)	9 (14.3)	84 (44.7)	
- Synchronous; n (%)	6 (9.5)	15 (8)	0.70
- Metachronous; n (%)	57 (90.5)	173 (92)	
- No extrapulmonary metastases; n (%)	41 (65.1)	130 (69.1)	0.08
- Treated extrapulmonary metastases; n (%)	19 (30.2)	57 (30.3)	
- Extrapulmonary metastases without treatment*;* n (%)	2 (3.2)	0 (0)	
- Extrapulmonary metastases, unknown treatment*;* n (%)	1 (1.6)	1 (0.5)	
Mean disease-free interval between primary tumor and pulmonary metastasis (metachronic metastases only) (mean ± SD) [month]	57.3 ± 72.2	47 ± 54.0	0.022*
Systematic lymph node dissection*;* n (%)	28 (44.4)	160 (85.1)	< 0.001*
Lymph node sampling*;* n (%)	10 (15.9)	17 (9)	0.13
Lymph node status			
- pN0; n (%)	37 (58.7)	148 (78.7)	< 0.001*
- pN1; n (%)	0 (0)	5 (2.7)	
- pN2; n (%)	1 (1.6)	23 (12.2)	
- pN3; n (%)	0 (0)	1 (0.5)	
- cN0; n (%)	25 (39.7)	11 (5.9)	
Postoperative stay (mean ± SD) [days]	6.7 ± 2.5	10.1 ± 4.9	< 0.001*
Required surgical revision*;* n (%)	0 (0)	9 (4.8)	0.08
In-hospital mortality; n (%)	0 (0)	1 (0.5)	0.56

*SD* Standard definition, *VATS* Video-assisted thoracoscopic surgery

*significance *p* < 0.05

### Operative data

In both groups, wedge resection was the most often performed procedure, followed by lobectomy (Fig. 1). Only patients who underwent thoracotomy via combinations of different types of resection were analysed. Application of the different types of resection showed no difference in either group (VATS vs. thoracotomy; *p* = 0.1).

In 89% (*n* = 223) of the patients, complete (R0) resection was obtained (VATS 90.5%, thoracotomy 88.3%). Resection was classified as R1 in 14 (5.6%) patients. Only in one patient in the thoracotomy group was macroscopic removal of the metastasis not feasible. Again, there was no significant difference in either group (*p* = 0.69). The average distance between the staple line and the tumour was 8.5 ± 9.1 mm (*n* = 57) in patients who underwent VATS after R0 resection.

Systematic lymph node dissection was performed significantly more often in the thoracotomy group than in the VATS group (*p* < 0.001). In some cases, only lymph node sampling was performed (VATS 15.9% vs. thoracotomy 9%; *p* = 0.13). According to pathological reports, stage pN0 represented most cases after VATS and after thoracotomy. All patients who did not undergo lymph node resection for histological confirmation were preoperatively in clinical stage N0 (cN0). The rate of patients with a lymph node status of cN0 was higher in the VATS group. Overall, there was a significant difference with regard to the histological lymph node status in both groups (*p* < 0.001).

### Postoperative outcome

The mean hospital stay was 6.7 ± 2.5 days after VATS and 10.1 ± 4.9 days after thoracotomy (*p* < 0.001). Postoperative revisions were necessary in only nine patients (4.8%) in the thoracotomy group. However, this number was still not sufficient to reach statistical significance (*p* = 0.08). Reasons for surgical revision were wound healing disorders (*n* = 3), chylothorax (*n* = 2), haemothorax (*n* = 1), prolonged parenchymal fistula (*n* = 1), anastomosis insufficiency (*n* = 1) and necrosis of pulmonary tissue (*n* = 1). One patient died of pulmonary embolism after thoracotomy (0.5%). This patient was the only reported death within the hospital stay after surgery in both groups, resulting in an in-hospital mortality rate of 0.4% (*n* = 1).

During follow-up, eight (12.7%) patients from the VATS group and 41 (21.8%) patients from the thoracotomy group experienced recurrence of pulmonary metastasis. Of these, two who underwent VATS (25%) and 12 who underwent thoracotomy (29.3%) underwent reoperation due to recurrent pulmonary metastases.

### Survival

**Fig. 3:**
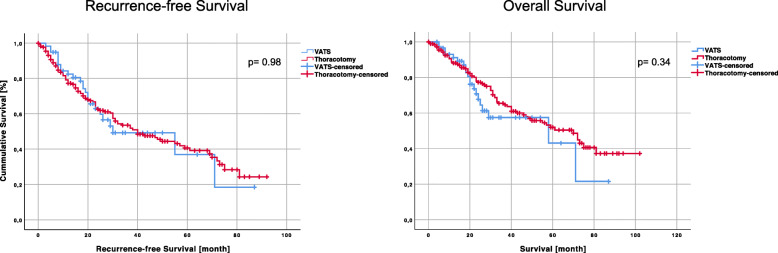
Recurrence-free survival (RFS) and overall Survival (OS) of all patients

Kaplan-Meier OS and RFS curves are shown in Fig. 3. Considering all patients, a median OS of 61 months (95% confidence interval 46.1–75.9) was observed. After VATS and thoracotomy, 5-year survival rates of 43.1 and 51.9% were obtained. There was no significant difference in survival regarding the surgical procedure (VATS: 58 months [95% confidence interval 3.1–112.9] vs. thoracotomy 70 months [95% confidence interval 50.4–89.6]; *p* = 0.34). The median overall RFS was 44 months (95% confidence interval 26.8–53.2). Patients who underwent VATS experienced a slightly shorter RFS (30 months) (95% confidence interval 6.1–53.6) than patients who underwent thoracotomy (40 months) (95% confidence interval 26.4–53.6). Additionally, in terms of RFS, there was no significant difference in the log-rank test (*p* = 0.98). To identify the prognostic impact, a multivariate Cox proportional hazards model was generated. Included variables were sex (*p* = 0.74), age (*p* = 0.07), procedure performed [VATS, thoracotomy] (*p* = 0.94), tumour location [central, peripheral, intermediate] (*p* = 0.68), resection status [R0, R1, and R2] (*p* = 0.35), occurrence of metastasis [synchronous, metachronous] (*p* = 0.16), lymph node status [cN0, pN0, pN1, pN2, and pN3] (*p* = 0.03) and systematic lymph node dissection performed [yes, no] (*p* = 0.15).

**Fig. 4:**
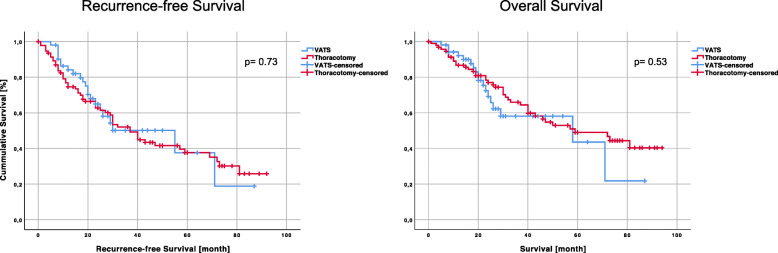
Recurrence-free survival (RFS) and overall Survival (OS) in patients with solitary metastasis

When patients with solitary metastasis only were considered, the median RFS after VATS and after thoracotomy was equivalent (VATS: 55 months [95% confidence interval 20.1–89.9] vs. thoracotomy: 37 months [95% confidence interval 28.4–45.6], *p* = 0.73). There were also no significant differences with regard to OS in these patients (VATS: 58 months [95% confidence interval 2.8–113.2] vs. thoracotomy: 59 months [95% confidence interval 32.4–85.6], *p* = 0.53) (Fig. 4).

## Discussion

Pulmonary metastectomy is the second most frequently performed procedure in thoracic surgery, and with 15–50% of the workload of thoracic surgeons, it represents a major part of their profession [18]. While minimally invasive techniques have been established for many indications of thoracic surgery, thoracotomy is still preferred for metastatic surgery with curative intent. Its advantage is the opportunity to palpate the lung intraoperatively [19]. However, the role of VATS and its influence on resection and long-term results remain unclear in times of constantly improving preoperative diagnostics [14, 16]. In this retrospective study, 251 patients who underwent curative metastectomy via VATS or thoracotomy were analysed to evaluate postoperative outcomes, including complications, mortality, resection status and survival.

The most frequent indication for pulmonary metastectomy in the literature is metastases of colorectal carcinomas [18]. This was also observed in our study, in which this tumour entity accounted for 37.8% of all patients. The majority of patients had a solitary metastasis, especially those who were treated with VATS. This difference was statistically significant (*p* < 0.001) and was certainly often a factor in the decision making of which procedure to perform. Both patients who underwent VATS and those who underwent thoracotomy mainly had metastases that were detected on average 47 to 57 months after successful therapy of the primary tumour (metachronous disease). Since this interval is a good indicator that can be used to describe the dynamics of the disease, the patients in both groups seemed to experience rather moderate progression, with an average disease-free interval of more than 4 years [20]. In contrast, synchronous metastasis is associated with a rather poor prognosis. In the case of a short disease-free interval or synchronous metastasis, primary chemotherapy should be recommended, and the proportion of such patients was therefore small in this study [21].

Complete resection is the most important factor regarding postoperative outcomes of metastatic surgery [11–13]. In this study, there was no detectable difference in the resection status of the pathological reports in either group. In patients who underwent VATS as well as in those who underwent thoracotomy, the majority of metastases were resected via R0 resection (VATS 90.5%, thoracotomy 88.3%). In the VATS group, the average distance between the staple line and the tumour after R0 resection was sufficient, at 8.5 mm (*n* = 57). For the remaining R1 tumours, it must be acknowledged that there was a small, additional safety margin between the staple line and surgical margin. In addition to the high rate of complete R0 resection, the fact that none of the patients had a postoperative R2 situation after VATS shows that minimally invasive procedures are safe and feasible when carefully selected preoperatively.

The therapeutic benefit of additional lymph node dissection in thoracic metastases remains the subject of intense discussion. The involvement of mediastinal lymph nodes or an unclear nodal status is associated with significantly decreased survival [22]. Systematic lymph node dissection can therefore provide important information to refine the estimation of the prognosis by determining nodular dissemination and complete staging [22–24]. In a survey among members of the European Society of Thoracic Surgeons (ESTS), most surgeons reported performing mediastinal lymph node sampling for an indication for possible adjuvant treatment. Approximately one-third reported neither sampling nor systematic dissection [8, 25]. Systematic lymph node dissection was performed significantly more often in patients who underwent thoracotomy in our study (*p* < 0.001). This may be because patients with multiple metastases or suspicious lymph nodes on imaging were preferably treated with an open procedure. In contrast, the rate of patients with a lymph node status of cN0 was higher in the VATS group, which certainly contributed to the decision to perform VATS.

As expected, the average postoperative stay after VATS was significantly shorter than that after thoracotomy. The reduced trauma and less pain caused by VATS have been demonstrated in other studies to result in faster postoperative recovery and shorter discharge times [26, 27].

Extrapulmonary metastases were detected in 80 patients (31.9%). The rate of extrapulmonary metastases as a sign of advanced disease was balanced in both groups, so no bias in further analyses must be assumed (VATS 34.9% vs. thoracotomy 30.1%). In the analyses of OS and RFS, no significant differences regarding the surgical approach was found. This may indicate that the impact of the procedure used on survival is small and that the greater trauma of thoracotomy should be considered carefully. Very few retrospective studies are available on this issue, and most focus on metastases of colorectal carcinomas [28]. The majority of them also suggest that VATS provides equivalent survival while having several known advantages over open procedures [17, 29, 30]. Nevertheless, many clinicians still choose thoracotomy or mini-thoracotomy for metastectomy. Further prospective work is required to confirm the non-inferiority of VATS in curative metastectomy concerning OS and RFS assuming that a particular preoperative evaluation (lymph node status, solitary metastasis) was carried out.

## Conclusions

VATS metastectomy should be considered, especially in patients with a solitary metastasis. In our study, histologically complete (R0) resection was achieved in 90.5% of the patients who underwent VATS and was thus comparable to the resection status after thoracotomy. VATS metastectomy was also shown to be equivalent to thoracotomy with regard to lymph node status. In addition, recurrence-free survival and overall survival were comparable to conventional, open resection of metastases. Therefore, an open surgical approach with palpation of the lung showed no advantage in terms of surgical outcome or survival.

### Limitations

First, the retrospective nature of this study must be mentioned. Since the observation period was long, there was certain evolution of the surgical approach. Thus, the rate of thoracotomies performed in this study was relatively high. Concerning the sample size of the two groups, the VATS group was smaller, resulting in a bias towards the VATS group. Certainly, the choice between VATS or thoracotomy was influenced by the number of preoperative metastases as well as the preoperative lymph node status.

## Data Availability

The datasets used and/or analysed during the current study are available from the corresponding author on reasonable request.

## Abbreviations

CT: Computed tomography
OS: Overall survival
RFS: Recurrence-free survival
VATS: Video-assisted thoracoscopic surgery
